# Material State Awareness for Composites Part II: Precursor Damage Analysis and Quantification of Degraded Material Properties Using Quantitative Ultrasonic Image Correlation (QUIC)

**DOI:** 10.3390/ma10121444

**Published:** 2017-12-18

**Authors:** Subir Patra, Sourav Banerjee

**Affiliations:** Integrated Material Assessment and Predictive Simulation Laboratory, Department of Mechanical Engineering, University of South Carolina, Columbia, SC 29208, USA; spatra@email.sc.edu

**Keywords:** precursor damage, material state awareness, composite, ultrasonic, damage entropy, scanning acoustic microscopy (SAM), NDE

## Abstract

Material state awareness of composites using conventional Nondestructive Evaluation (NDE) method is limited by finding the size and the locations of the cracks and the delamination in a composite structure. To aid the progressive failure models using the slow growth criteria, the awareness of the precursor damage state and quantification of the degraded material properties is necessary, which is challenging using the current NDE methods. To quantify the material state, a new offline NDE method is reported herein. The new method named Quantitative Ultrasonic Image Correlation (QUIC) is devised, where the concept of microcontinuum mechanics is hybrid with the experimentally measured Ultrasonic wave parameters. This unique combination resulted in a parameter called Nonlocal Damage Entropy for the precursor awareness. High frequency (more than 25 MHz) scanning acoustic microscopy is employed for the proposed QUIC. Eight woven carbon-fiber-reinforced-plastic composite specimens were tested under fatigue up to 70% of their remaining useful life. During the first 30% of the life, the proposed nonlocal damage entropy is plotted to demonstrate the degradation of the material properties via awareness of the precursor damage state. Visual proofs for the precursor damage states are provided with the digital images obtained from the micro-optical microscopy, the scanning acoustic microscopy and the scanning electron microscopy.

## 1. Introduction

Currently, the understanding of the precursor damage state in composites relies predominantly on the multi-scale composite failure models [1,2,3,4]. Experimentally, on the other hand, the ultrasonic sensing (nondestructive evaluation (NDE)) methods [5,6,7], X-ray tomography the digital image correlation (DIC) [8] methods are used to understand the material states. However, there has been a persistent disconnect between the sensing and the modeling efforts. The damage tolerance design of the structural composites is currently based no-growth criteria, which is economically unrealistic. To enable the slow growth criteria in the damage tolerance design of composite, it is necessary to incorporate the understanding of the precursor damage state. Although the change in ultrasonic Leaky Lamb wave velocities due to matrix cracking [9] was reported, during the service life, there is no quantified process to compromise the material properties in the virtual models, predictively. Rather currently the anisotropic constitutive material properties are progressively compromised by an arbitrary percentage value. Such failure models usually result in erroneous remaining strength of the material. They are divergent from the experimental results, reported by many leading composite users and manufacturers. The objective of this article is to aid the progressive failure models with a predictive material degradation parameter derived from an ultrasonic NDE method. In the next paragraph, possible precursor states in composites and their interactions with the ultrasonic NDE method are discussed.

### 1.1. Definition of Precursor

Precursor damages in the composite develop in the form of matrix cracking, microcracks, voids, micro-buckling, local fiber breakage, local fiber-matrix deboning, interlaminar shear, etc. The precursor state starts to occur during the first 10% of the composite life. In low frequency (<~10 MHz) NDE experiments the signal decay, signal delay, phase shifts and the overall wave annihilation (due to attenuation and scattering) are the predominant features [9] that are extracted from the ultrasonic signals to quantify the damages (cracks and delamination) in the composite structures. However, the contribution of the precursor damages to these features might be insignificant or undetectable using guided Lamb wave modes. During the first stage in the life cycle of the composites, even before forming the above-mentioned damages, material state changes with the changes/degradation/reconfigurations of the local material properties. Distributed stress concentration and redistribution of the manufacturing defects also lead to these precursor states. The precursor damage states are neither localized nor static, but rather distributed and changes their forms by reorientation and reconfiguration of the material properties. 

It was found that the distributed precursor damage in the composite happens in a sequence of discrete events. Further, these embryonic damages distribute themselves until the next precursor event happens. Next, after the 50% of the composite life, an avalanche effect of many precursor events creates the discrete damage in the composite. The duration between two precursor events can be compared with the dynamic relaxation of composites after loading. This article provides the opportunity to further study this phenomenon in detail.

### 1.2. QUIC Using Nonlocal Mechanics for Material State Awareness

To predictively conclude the material state during the precursor, the microcontinuum mechanics based features hybrid with the high frequency ultrasonic (>~25 MHz) is proposed in this article. With previous research [10,11] it was found that the high frequency (>~25 MHz) Ultrasonic wave, propagated through the composite material, interacts with the precursor damages and potentially carries the features that are linked to the local degradation of the material properties. However, these hidden features are subtle and sometimes confusing to make a conclusion. Opposing the existing norms Quantitative Ultrasonic Image Correlation (QUIC) is devised with the help of high-frequency Scanning Acoustic Microscopy (SAM) [12] and nonlocal mechanics. Acoustic microscopy was previously used for quantifying the residual stress [13] and determining the local mechanical properties [14,15,16]. However, was never utilized for the precursor damage state quantification. Here in the QUIC, the wave signal at each pixel of an ultrasonic image is analyzed in the context of nonlocal mechanics.

Nonlocal mechanics [17] considers the effect of all the neighboring material points on a parent material point of interest, unlike the continuum mechanics, where the constitutive law is valid only at one point. In the precursor state in a distributed sense, the neighboring material points interact with each other to reconfigure/reorient themselves to relax the local stress concentrations, loosening or hardening the material. Previously nonlocal mechanics [17] was studied for failure analysis but was never studied for quantifying precursor damage state. In the following paragraph, the material state awareness about the precursor damage states is discussed. 

Material state awareness with precursor is knowledge-based information about a state of the material that is not necessarily damaged with respect to the macro scale interpretation of the cracks/delamination. During the operation, due to compromised material properties at the lower scales, there is a potential for generating the distributed damages. Material state awareness should be a scale-dependent concept. Say for example a material with a crack or delamination with a 1 mm size (macroscale) has a larger footprint of damage around that 1 mm x 1 mm pixel area at the lower scale (microscales). It can be argued that when discrete macroscale damage is detected by a conventional nondestructive evaluation (NDE) method, several microscale damages have already taken place in the material in a distributed sense. Currently, such damage state cannot be quantified. Material properties are degraded even far away from that discrete damage sites. As mentioned before, such states are non-static, dynamic or/and rather chaotic. 

### 1.3. Quantification of Degraded Material Properties

Based on the above discussions, it can be said that the precursor damage state of the material is a spatiotemporal function of the progressive damage state of the material which cannot be predictively incorporated in the progressive failure models. Here, for the awareness of the material state, an attempt has been made to quantify this chaotic state of a material by calling the ‘precursor damage state’, such that an appropriate quantified parameter can be used to sacrifice the material properties in progressive composite failure models. The damage mechanics approach always had an advantage over other approaches [18]. In the damage mechanics based approach, damage tensors are used to degrade the local material properties [19,20], and failure mechanisms are introduced relating the damage variables to the strain energy release rate. Damage development laws depend on few failure criteria, such as exceeding the local stress above a specified, designed value. These criteria are based on the material parameters used in the failure equations, which depends on the material characteristics that are either heuristically measured from the specimen load tests or just randomly assumed. This article may provide an opportunity to rethink these criteria in the progressive failure models where quantified damage parameter called ‘damage entropy’ can be used to sacrifice the material properties, predictively as opposed to the current random processes. In the following paragraph, the term entropy is explained in the light of the precursor damage state.

Entropy is a mathematical definition that is associated with a naturally changing chaotic system. Entropy measures the degree of disorder or the randomness of an inherent property of a system. When a composite material is subjected to fatigue loading, at different locations of the composite, the local stiffness changes randomly due to the microstructural reorientation and the development of distributed damage. This process is irreversible and hence, could be defined in terms of an increasing entropy at a given time point, that represents the cumulative sum of the probability of different possible states of the system. To quantify the change in this chaotic material system, quasi-longitudinal wave velocity (along thickness) is calculated at each pixel point using acoustic microscopy. Thereafter, nonlocal parameters are calculated from the theoretical dispersion curves. Nonlocal parameters were then used to calculate an irreversible quantified parameter that is tied to the damage information and hence the ‘damage entropy’ term is introduced.

### 1.4. The Proposed Study

In this study, the QUIC is proposed with very high frequency ultrasonic between ~25 MHz–~100 MHz because the high-frequency waves are sensitive to the small-scale damages. First, the high-frequency SAM images with the wave velocity data recorded at each pixel point are correlated. Then QUIC tracks every pixel [11,21] over the total loading period and quantifies the chaotic state that evolves in the composite specimens. Additionally, to confirm the visualization of the fluctuation in the nonlocal parameter, Micro-optical microscopy using Keyence VHX-5000 series digital microscope (Itasca, IL, USA) was used. It was found that over the course of the material life, initially few pixels on the material were comparatively more compromised in terms of wave velocity than the other pixels. With the QUIC the compromised pixels were identified as local areas with precursor damages and was verified with the micro-optical microscopy images. Following the trend of the damage sites, after the 30% of the composite life, damage initiation was identified using SEM on a decommissioned specimen, where the internal precursor damage states were explicitly visualized.

## 2. Materials and Methods

### 2.1. Composite Fatigue Testing

**Figure 1:**
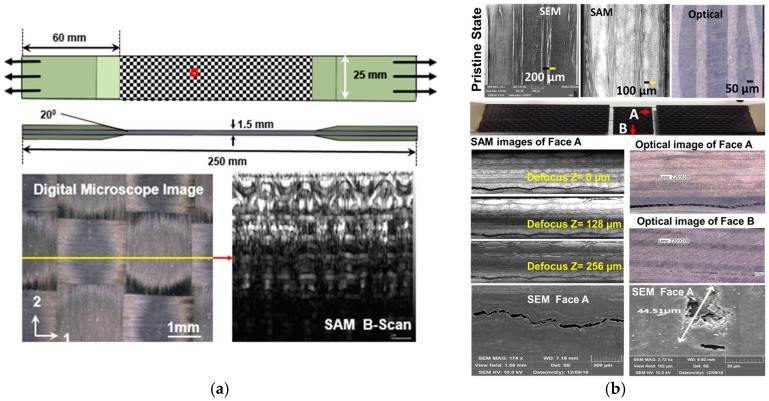
(**a**) Schematic of specimen geometry: Pristine internal structures are shown by digital microscope and scanning acoustic microscope; (**b**) Damages in woven composite specimen observed after ~2 million cycles, delamination started after ~1 million cycles.

In this study four-layer woven carbon-fiber composite material was used. The thickness of each lamina was 280 μm. A 2D woven structure is shown in Figure 1. Dimensions of the specimens are chosen according to the American Standard for Testing and Material standard, ASTM D 3039 [22], as shown in Figure 1a. The average length, width and thickness of the specimens were ~249.7 mm, ~24.7 mm, ~1.5 mm.

A total of eight specimens (T1, T2, T3, F-L, S-A, S-E, S-F, and S-G) were prepared. Three specimens T1-T3 were tested under pure tensile test using MTS 810 to estimate the ultimate load. The average ultimate load was 8200 lbf or 36.43 kN. Further only ~50% of the maximum load was used to create the tensile–tensile fatigue sequence with a load ratio R = 0.01 (R = F_min_/F_max_). The specimen F-L was used to estimate the fatigue life of the composite. The F-L specimen did not fail until ~2 million cycles, but significant damages were observed when it was placed under the light microscope, SAM and SEM (Figure 1b).

Delamination was first observed at the end of ~1 million cycles, marked as the fatigue life of the material. ~30% of that fatigue life, i.e., 300,000 cycles of fatigue loading were further considered for precursor damage analysis in the remaining four (4) specimens (S-A, S-E, S-F, and S-G). S-E, S-F, and S-G were used for offline precursor damage detection using QUIC, S-A specimen was tested in the similar loading environment and was decommissioned at the end of 300,000 cycles. SEM was performed to visualize the precursor indications. During the fatigue testing until the 300,000 cycles, the experiments were stopped every 10,000 cycles. QUIC and micro-optical microcopy imaging were performed at every interval on the S-E, S-F, and S-G specimens.

### 2.2. Experimental Process for QUIC

**Figure 2:**
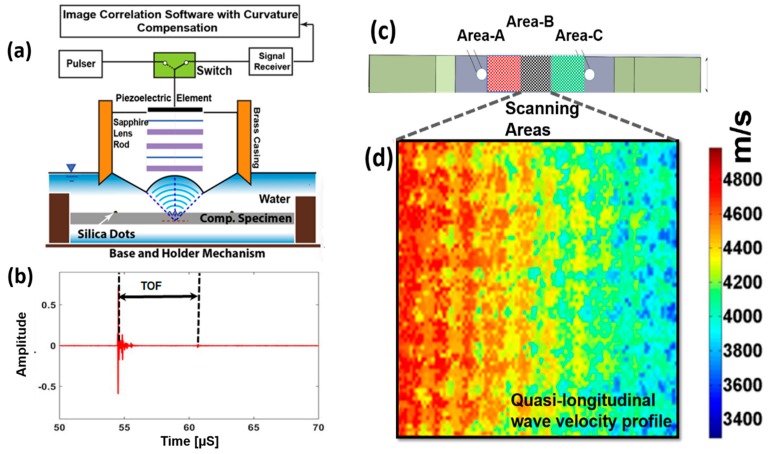
(**a**) Schematic of Scanning Acoustic Microscopy (SAM); (**b**) A typical A-Scan signal at a pixel point; (**c**) scanning areas on the specimen; (**d**) quasi-longitudinal wave velocity profile on a selected area.

Gage areas on the specimens S-E, S-F, and S-G, were scanned using the SAM (25 MHz, 50 MHz, and 100 MHz). Gage area of each specimen was divided into three different zones, Area-A, Area-B, and Area-C, respectively as shown in Figure 2. The specimens were submerged in to the water. Scanning probe was lower to the water level to focus the transducer and the scans were performed. Scans were performed every 10,000 cycles until the scheduled 300,000 cycles using ~25 MHz focused lens. To visualize the degraded material properties or development of micro-cracks across the depth of the specimens, ~50 MHz, and ~100 MHz transducers were used to perform the C-scans and X-scans. More details on this technique can be found in the operation principles of scanning acoustic microscopy (SAM) [23,24].

### 2.3. Benchmark Study Using Micro-Optical Imaging

Digital Microscopy (VHX-5000 series, Keyence Corporation of America, Itasca, IL, USA) imaging was performed at the interval of 10,000 cycles up to 300,000 cycles. Specimens were viewed at different magnifications between 0.1× and 50×, nondestructively. Similar to the QUIC, Area-A, Area-B, and Area-C were selected, and images were taken every 10,000 cycles.

## 3. Theoretical Development of QUIC

### 3.1. Damage State Quantification Using Nonlocal Continuum Theory

In problems where long-range forces exist, the nonlocal interaction between neighboring material points prevail. For example, relaxation of material properties, damage reconfiguration, relaxation and regeneration of stress concentrations, are few examples of such states where nonlocal interactions could be presumed. To investigate the material state using high-frequency wave propagation, the constitutive law from continuum mechanics is not enough. Hence, a suitable kernel function was used to modify the constitutive law. The Christofell’s equation was modified using the nonlocal constitutive law, and the Eigenvalue problem was solved to obtain the nonlocal dispersion curves for different wave modes (quasi-longitudinal and quasi-shear) as functions of nonlocal parameters. Experimentally measured wave velocities were used to calculate the nonlocal parameters from the dispersion curves. Parametric variations of the nonlocal parameters were used to quantify the precursor state. A detailed discussion on this technique for damage state quantification can be found in references [25,26]. The basic formulation is briefly discussed herein.

According to the continuum theory, stress-strain relation can be written at a point *x* in a body Ω as follows

(1) $$\sigma_{ij}\left(x\right)=C_{ijkl}\left(x\right)\varepsilon_{kl}\left(x\right)$$

where $C_{ijkl}\left(x\right)$ are the constitutive material properties at a point *x*. By employing the nonlocal approach, stress-strain relation at a point $y\left(x_{n}\right)$ in the material body is modified by introducing the nonlocal kernel function as follows,

(2) $$t_{ij}(y)=\int_{\Omega }C_{ijkl}(x)H\left(\left|y-x\right|\right)\varepsilon_{kl}(x)d\Omega$$

where, H(|y−x|) is the nonlocal kernel function. By substituting the non-local constitutive law in the equation of motion, the integro-differential form of the equation at the point *y* can be written as,

(3) $$\int_{\Omega }\frac{\partial }{\partial x_{j}}\left(\sigma_{ij}(x)H\left(\left|y-x\right|\right)\right)d\Omega (x)+F_{i}(y)=\rho (y){\overset{¨}{u}}_{k}\delta_{ik}$$

Equation (3) is modified by using an operator *L*. The operator *L* is defined to make the kernel function a Green’s function.

(4) $$C_{ijkl}\varepsilon_{kl,j}(y)-L\left(\rho \left(y\right){\overset{¨}{u}}_{{}_{k}}\right)\delta_{ik}=-LF_{i}(y)$$

Bi-Helmholtz type operator *L* can be written as Lazar et al. [27]

(5) $$L=(1+\tau_{0}{}^{2}\ell^{2}\nabla^{2}+\nu_{0}{}^{4}\ell^{4}\nabla^{4})$$

where, $\nabla^{2}$ is a Laplace operator, τ and λ are the intrinsic length scale parameters, $\tau =\tau_{0}\ell \;\mathrm{and}\;\nu =\nu_{0}\ell$. The homogeneous nonlocal Christoffel equation is viz.,

(6) $$\left[C_{ijkl}\frac{\partial^{2}u_{k}(y,t)}{\partial x_{j}\partial x_{l}}-\rho \left(y\right)L\left({\overset{¨}{u}}_{k}(y,t)\right)\delta_{ik}\right]=0$$

By substituting the displacement function, $u_{k}=A_{k}\exp(ik.x-i\omega t)$, in the Equation (6), viz.

(7) $$\left[{\left|k\right|}^{2}\frac{T_{ik}}{\rho \left(x\right)\omega^{2}}-\left(1+\tau_{0}{}^{2}{\left|k\right|}^{2}-\nu^{4}{\left|k\right|}^{4}\right)\delta_{ik}\right]=0$$

where, ${\left|k\right|}^{2}=(k_{1}^{2}+k_{2}^{2}+k_{3}^{2})$, defining, $\Omega_{ik}=\frac{T_{ik}}{\rho \left(x\right)\omega^{2}}$, Equation (7) is rewritten as follows,

(8) $$\left[(\Omega_{ik}-\tau_{0}{}^{2}\delta_{ik})-\left(\frac{1}{{\left|k\right|}^{2}}-\nu^{4}{\left|k\right|}^{2}\right)\delta_{ik}\right]=0$$

By solving the Eigenvalue problem, the dispersions of the wave modes are obtained for different $\tau_{0}$ parameters. Only the positive roots of the equation were considered. The nonlocal parameter $\nu_{0}$ was first introduced by Lazar et al. [27] to influence the dispersion relation further down the scale with a condition $\tau_{0}>\;\sqrt{2}\nu_{0}$. In this study a smallest non-zero value of $\tau_{0}$ was arbitrarily selected and was assumed to be 0.0017. Applying the condition given in reference [27] $\nu_{0}$ was calculated to be 0.0012 and was kept constant in this study. Next, the dispersion of the quasi-longitudinal wave mode in woven carbon-fiber composite specimens was calculated using the Equation (8). Material properties (obtained from the vendor, listed in Ref. [28]) were used to calculate the wave velocities are listed below.

$$\left[\begin{array}{cccccc} 81.64 & 27.74 & 27.74 & 0 & 0 & 0\\ 27.47 & 76.98 & 15.51 & 0 & 0 & 0\\ 27.74 & 15.51 & 76.98 & 0 & 0 & 0\\ 0 & 0 & 0 & 5 & 0 & 0\\ 0 & 0 & 0 & 0 & 5 & 0\\ 0 & 0 & 0 & 0 & 0 & 5 \end{array}\right]\;\mathrm{GPa}$$

**Figure 3:**
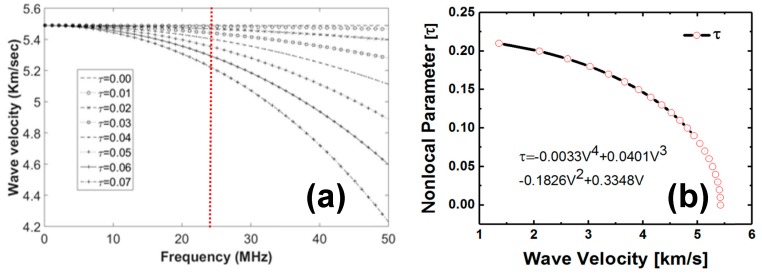
(**a**) Dispersion of quasi-longitudinal wave mode in carbon-fiber composite specimen; (**b**) variation of the nonlocal parameter at ~25 MHz.

The dispersions of the wave velocities obtained from the nonlocal Christoffel equation depend on the frequency and the nonlocal parameters presented in the Figure 3a. Further to find the relation between the change in the wave velocity concerning the nonlocal parameters at a fixed frequency (here ~25 MHz), a nonlocal-wave velocity plot is created as shown in Figure 3b. Through regression analysis, a mathematical equation for the relation between the nonlocal parameter and the quasi-longitudinal wave velocity in the composite material was obtained. Further, to calculate the nonlocal parameter from the equation, experimentally measured wave velocities (Figure 2) were used. The step-by-step processes to calculate the quasi-longitudinal wave velocity across the thickness of the composite specimen and the quantification of the precursor state at different fatigue interval are discussed below.

**Figure 4:**
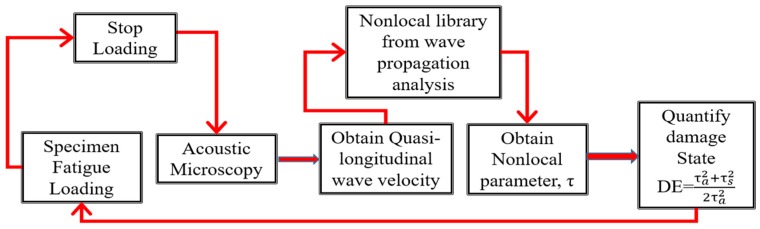
Process flow diagram showing the steps for damage quantification using nonlocal physics.

(1) Z-scans, where SAM scans were performed at different defocus distances across depth using a broadband ~25 MHz transducer manufactured by PVA Tepla AG, Wettenberg, Germany. Scans were performed at three different areas (Area-A, Area-B, and Area-C) on the specimen to cover the whole gage area as shown in the Figure 3c. Each area was discretized into a number of pixels, and the A-scan signal at each pixel point was recorded for further analysis.
(2) A typical A-scan signal at a pixel point is shown in the Figure 3b. The first wave packet in the signal is the normal reflection of the incident wave while the second one is the backside reflection of the transmitted wave.
(3) Time-of-flight (TOF) between the front and backside reflections can be seen as the time taken by the transmitted wave to travel through the specimen. TOF was calculated at each pixel point. By dividing the wave path (twice the thickness) of the specimen by TOF, quasi-longitudinal wave velocity at each pixel point was obtained. Then the 2D distributions of the wave velocities were obtained at three different scanning areas as shown in the Figure 2c.
(4) Next the nonlocal parameter, *τ*, were obtained from the nonlocal dispersion curve at ~25 MHz as shown in Figure 3b at each pixel.
(5) The nonlocal parameter obtained at the pristine state is called the intrinsic material property adjuster, $\tau_{a}$, while the parameter calculated at different fatigue interval is called the intrinsic material state parameter, $\tau_{s}$. Next, the average and the standard deviation of the intrinsic material state parameters were calculated from the data.
(6) The overall damage state of the material was quantified by the Nonlocal Damage Entropy (NLDE), using the above mentioned nonlocal parameters calculated at each pixel
  (9) $$NLDE=\overset{N}{\underset{i=0}{\sum }}{\left|\frac{\tau_{a}{}^{2}+\tau_{s}{}^{2}}{2\tau_{a}{}^{2}}\right|}_{i}$$
  where *i* denotes the *i*-th pixel on the specimen, and the NLDE is the summation of the nonlocal parameters (Equation (9)) over all the pixel points.
(7) Cumulative damage growth was then calculated to obtain the damage growth under the fatigue loading. The detailed process is shown in Figure 4.

### 3.2. Damage State Quantification from the Evaluation of Stiffness Degradation

In this study, the quasi-longitudinal wave velocity was measured at each pixel point along the thickness (direction-3) direction. The pixel points are distributed over the scanning areas Area-A, Area-B, and Area-C as described in the previous section. At a regular fatigue interval, the velocity profiles were obtained from the measured quasi-longitudinal wave velocity at each pixel point. Damage tenor was then calculated using a wave slowness model similar to the stiffness degradation model described in reference [29].

(10) $$\begin{array}{l} D_{ii}=1-\frac{C_{ii}}{C_{ii}^{0}}\\ i=1,2,\ldots ,6 \end{array}$$

(11) $$\begin{array}{l} D_{ij}=\frac{C_{ii}^{0}-C_{ij}}{C_{ij}^{0}+sign(C_{ii}^{0}-C_{ij}).\sqrt{C_{ii}C_{ij}}}i¹j\\ i=1,2,\ldots ,6\\ j=1,2,\ldots ,6 \end{array}$$

where *C_ij_* is the stiffness tensor. To calculate the degradation of the material properties across the thickness of the specimen, stiffness component (*C*_33_) in Equation (11) was replaced by the measured quasi-longitudinal wave velocity as follows,

(12) $$D_{33}^{N}=1-\frac{qL_{33}^{N}}{qL_{33}^{0}}$$

where, $qL_{33}^{0}$ is the quasi-longitudinal wave velocity at the pristine state of the composite specimen along the direction-3, and $qL_{33}^{N}$ is the wave velocity after *N*-th fatigue cycle in the same specimen. Then the cumulative damage growth was calculated as,

(13) $$DI=\overset{N}{\underset{k=1}{\sum }}D_{33}^{k}$$

Results are presented and discussed in the Result and Discussion section.

### 3.3. Probability Distribution of Quasi-Longitudinal Wave Velocity

To investigate the effect of degradation of material properties on the probabilistic distribution function for the wave velocity pattern, quasi-longitudinal wave velocity profile was obtained from the pristine state specimens. Next, at the end of each fatigue interval, a probability density function that best explains the distribution of the wave velocities over the areas Area-A, Area-B, and Area-C, collectively, were calculated. It was found that the degradation of the mean stiffness, between the pristine state and the state, at the end of 110,000 cycles are not negligible, which is conventionally assumed unchanged, during the first 10% of the life of a composite. This affirms that the reduction of the quasi-longitudinal wave velocities is random over the material surface. These reductions are distributed in nature. This signifies that the material properties started to compromise as early as 10% of the fatigue life of the composite specimens when the QUIC was able to indicate the initiation of precursor state. 

## 4. Results and Discussion

With the studies discussed above, it was confirmed that it is possible to detect and quantify the precursor damage state using the proposed technique. In the following subsections, findings from all the methods stated above are discussed.

### 4.1. Damage Quantification Using Quantitative Ultrasonic Image Correlation (QUIC)

#### 4.1.1. A Proof of Material Degradation

**Figure 5:**
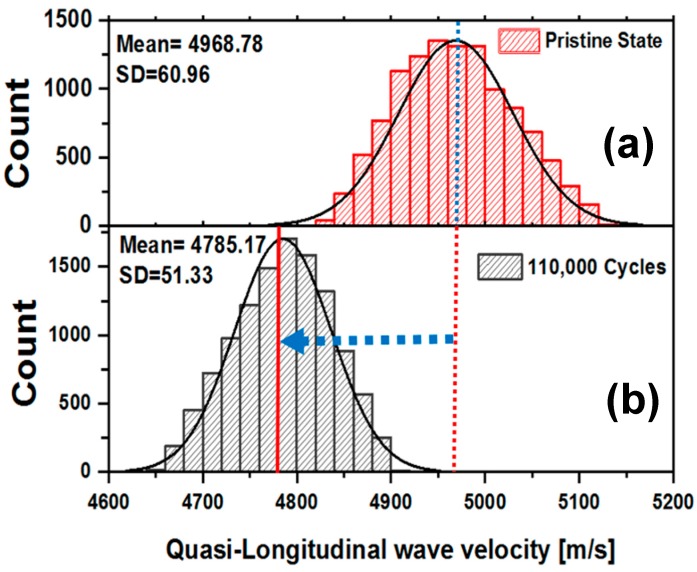
Probability density distribution of wave velocities. (**a**) Pristine state; (**b**) 110,000 cycles.

As described in Section 3.3 the probability distribution of quasi-longitudinal wave velocities was analyzed in specimen S-E at the pristine state and at the end of 110,000 cycles (Figure 5), which is at the end of 10% of the life of the composite. To find, if the distributed damages are developed inside as well as on the surface of the specimens, QUIC was performed on 125 × 125 pixels. Probability density function that best explains the distribution of the wave velocity over the areas Area-A, Area-B and Area-C were calculated by using MATLAB Statistical toolbox (R13, Mathworks, Natick, MA, USA). It was observed that the distribution of the quasi-longitudinal wave velocity was significantly altered.

The mean velocity and the standard deviation of the wave velocity profile changed due to the distributed precursor damage inside the specimen. The standard deviation of the quasi-longitudinal wave velocity distribution was decreased from the pristine state to the end of 110,000 cycles, indicative of a reduction of the broader distribution of the local material properties in the specimen. It is argued here that the wave velocity has decreased due to the reconfiguration of the material points, i.e., reconfiguration of local stiffness and/or density. When the material is in the pristine state, there were several manufacturing defects, and the material had local stress concentrations which were relaxed due to the initial set of fatigue loading. Overall the material state was going towards a converged state, but only by compromising the material properties. However, on the contrary, according to the definition of the entropy from statistical mechanics, it is the sum of all the possible ways a system can be taken back from its current state to the original state, which always increases. By that definition, each material point in the specimen has actually diverged, from its original pristine state to the current state, with inevitable increase in the entropy of the system. This is indeed a challenging new argument to perceive, however, true, further which is under study to be conclusively verified.

#### 4.1.2. Damage Quantification Using Nonlocal-Continuum Mechanics

**Figure 6:**
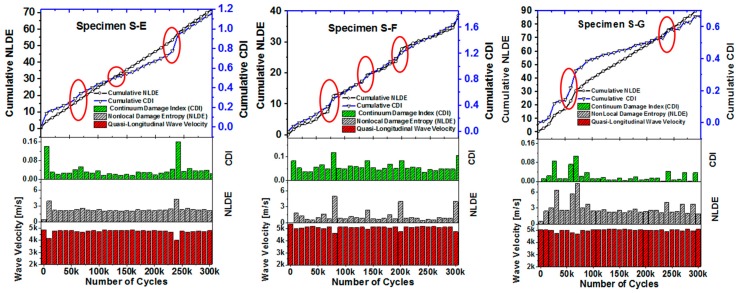
The data shows the cumulative growth of damage entropy quantified by QUIC. Sudden change is gradient in the NLDE are the indication of precursor damage event which tends to get distributed until the next event occurs.

As described in Section 2, in three composite specimens S-E, S-F, and S-G, the damage development stages were studied using the QUIC. Quasi-longitudinal wave velocity along the thickness directions at each pixel was calculated covering the scanning areas, Area-A, Area-B, and Area-C, respectively. Each scanning area was discretized into 125 × 125-pixel points, and wave velocity was calculated at each pixel point. At the pristine state, the average quasi-longitudinal wave velocity obtained from the specimens S-E, S-F, and S-G were ~5057 m/s, ~5171 m/s, and ~4959 m/s, respectively. However, the quasi-longitudinal wave velocity calculated after 300,000 cycles were ~4950 m/s, ~4754 m/s, ~4796 m/s, respectively. Precursor damage state in the specimens was quantified by plotting the cumulative Nonlocal Damage Entropy (NLDE) described in Section 3.1 (Figure 6). Although the increasing trend of the NLDE growth pattern is promising, it is relevant to focus on the incremental changes in the NLDE (bar charts in Figure 6) which were observed consistently in all three specimens near similar fatigue intervals. In fact, the sudden jumps (explained in Section 4.1.3) in NLDE and CDI were evident after ~70,000, ~110,000 and ~240,000 cycles. These are the states when material switched its state from one form of damage to the others. As per the Section 4.1.1 at the end of 110,000 cycles, i.e., after ~10% of the specimen life, material properties degraded and at the end of 240,000 cycles, i.e., after ~25% of the specimen life, micro-cracks were evident.

#### 4.1.3. Benchmark Damage Quantification from Stiffness Degradation

As described in Section 3.2 the continuum damage index (CDI) from the degradation of the material properties, the equivalence of material stiffness in terms of the quasi-longitudinal wave velocity was calculated at each pixel on the specimen. As QUIC was employed with high-frequency ultrasonic testing, it is expected that the CDI be qualitatively consistent with the NLDE for indicating the precursor damage in the specimens. In Figure 6, both the CDI and the NLDE for the specimens S-E, S-F and S-G are plotted to facilitate the discussion on identifying the precursor damage state that initiated in the specimens. In Figure 6a–c it was observed that the CDI indicated cumulative damage growth with fatigue cycles, the precursor events were identified in very close proximity to the similar fatigue intervals indicated by the NLDE independently, which were between ~50,000 to ~80,000 cycles, ~110,000 to ~175,000 cycles and ~225,000 cycles to ~280,000 cycles, inclusive all methods and all specimens. It is concluded that the material type tested initiated the precursor damage within ~30% of its lifespan. 

In this section further it is recommended, what percentage of material properties should be degraded during the progressive failure model tested under fatigue. It is proposed to mark a threshold value (dotted red line in Figure 6) of the NLDE based on the Student t-distribution analysis and identify the outlier, which is defined as the jumps in the NLDE and the CDI plots. Next, the value of the outlier NLDE occurred at the end of the respective fatigue interval should be used to compromise the material properties. For example, as per Figure 6b, in specimen S-F, it is recommended to compromise the material properties by 3.5% at the end of 200,000 cycles. The new constitutive material property tensor can be written as,

(14) $${\left(C_{ijkl}\right)}_{200,000}={\left(C_{ijkl}\right)}_{0}-\left[{\left(C_{ijkl}\right)}_{0}*{\left(NLDE\right)}_{200,000}/100\right]$$

### 4.2. Benchmark Damage Characterization Using Micro-Optical Microscopy

Optical microscopy imaging was performed on the composite specimens to examine the precursor damage. In the pristine state manufacturing defects were present in the specimens in the form of local voids with size ~6 ± 1 µm. However, it is evident from the microscopy images that the density of the microstructural damages increased due to the fatigue loading. Matrix cracking, fiber breakage, and localized inter-laminar delamination are observed at the end of ~160,000 and ~300,000 cycles. The average size of the matrix-cracks was observed close to ~224 μm. 

**Figure 7:**
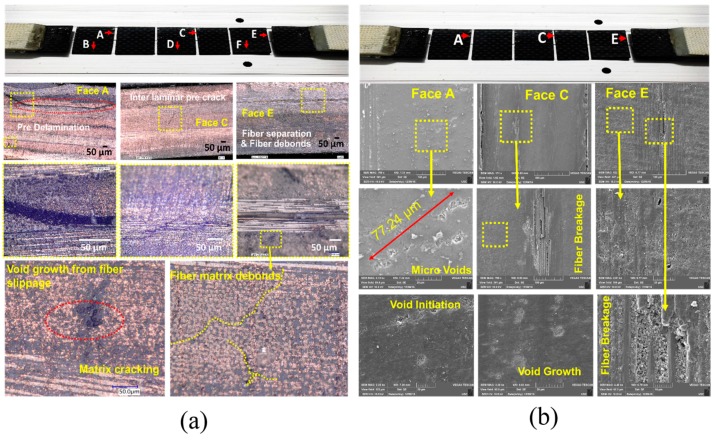
(**a**) Optical microscopy images of the decommissioned specimen S-A at the end of 300,000 cycles; (**b**) Scanning Electron Microscopy (SEM) images from the decommissioned specimen S-A after 300, 000 cycles of fatigue loading.

Large-scale damages such as edge delamination were not observed in the specimens. To investigate the development of the precursor damages across the width, at the end of 300,000 cycles specimen S-A was decommissioned and was cut at three locations (Figure 7), carefully using waterjet machine. Face A, B, and C, were then ground up to 3 mm by using P1200 sandpaper. Afterwards, all faces were polished with P2400 sandpaper to get a smooth surface. Pre-delamination, fiber separation, and fiber debond, voids from the fiber slippage and interlaminar delamination crack joining two adjacent matrix cracks [1] are evident in the specimen S-A (Figure 7).

### 4.3. Damage Characterization Using Scanning Electron Microscopy (SEM)

Face A, C and E were further investigated using SEM VEGA3 (TESCAN, Brno-Kohoutovice, Czech Republic), and a summary of the findings is presented in Figure 7. Working distance in SEM was 7.22 mm and the accelerating voltage was 10 kV. Multiple sites of void initiation, the existence of large voids, fiber breakage, were identified and they confirm the findings from the benchmark studies. 

### 4.4. Damage Characterization Using Scanning Acoustic Microscopy (SAM)

**Figure 8:**
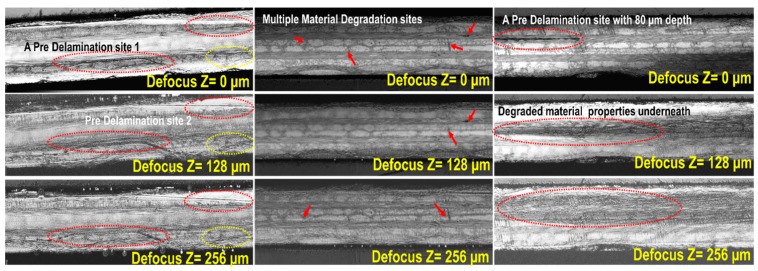
Scanning Acoustic Microscopy (SAM) images from the decommissioned specimen S-A after 300,000 cycles of fatigue loading.

SAM was performed on the specimen to investigate the damage developments on the surface as well as inside the specimens which were not accessible by the micro-optical microscopy. SAM C-scans were performed using high resolution ~100 MHz ultrasonic transducer at three defocused distances (Figure 8) at three locations, Face A, C and E. Matrix cracking was clearly visible on the surface of the specimens. A couple of delamination sites were also observed. Additionally, from the grayed zone with lower wave amplitude, degraded materials properties were observed beneath the pre-delamination site. Multiple immature interlaminar delamination tracks were observed joining two matrix cracks or matrix fiber disbond tracks.

## 5. Conclusions

The objective of this article is to aid the progressive failure model with a quantified physics based parameter to predictively degrade the material properties of the composites under fatigue. Hence, a hybrid nonlocal mechanics based offline ultrasonic NDE method is devised to quantify the material degradation. In this work, four woven fiber composite specimens were tested under high cycle low load fatigue loading to develop the progressive damage inside the specimen within their 30% of life calculated to be more than 300,000 cycles. The QUIC was used to measure the wave velocities on the gage sections of the specimen to transform the information to a nonlocal parameter calculated from the dispersion curve obtained from the nonlocal Christoffel equation. Damage growth was quantified by nonlocal damage entropy (NLDE). Continuum damage index (CDI) across the thickness of the specimen was also quantified by the stiffness-degradation method. Cumulative damage growth was plotted with the number of fatigue cycles. The probability distribution of the degraded wave velocity over the specimen was plotted at the pristine state and at the end of ~110,000 cycles. This unique and consistent phenomenon will help devise new damage detection algorithm for online precursor damage detection and quantification. Further using the outlier NLDE parameter, the recommendation is given, how to sacrifice the material property tensor during the virtual fatigue testing in the simulation environment.

## Abbreviation

$\sigma_{ij}$	Stress Tensor
$\varepsilon_{kl}$	Strain Tensor
$C_{ijkl}$	Constitutive Matrix
$t_{ij}$	Nonlocal Stress Tensor
H(|y−x|)	Nonlocal Kernel function
F	Force
ρ	Density
$u_{k}$	Displacement
$\delta_{ik}$	Kronecker delta
L	Laplace operator
$A_{k}$	Amplitude Vector
k	Wave Vector
ω	Frequency
τ	Intrinsic length scale factor
ν	Intrinsic length scale factor
l	Length Scale
$\tau_{0}$	Nonlocal Parameters
$\nu_{0}$	Nonlocal Parameters
$\tau_{a}$	Intrinsic material property adjuster
$\tau_{s}$	Intrinsic material property state
$c_{ij}$	Stiffness tensor
$qL_{ij}$	Quasi-longitudinal wave velocity
$D_{ij}$	Damage Tensor
